# In response to Fogarty et al. and why adjuvant whole brain radiotherapy is not recommended routinely

**DOI:** 10.1186/s12885-017-3672-z

**Published:** 2017-11-15

**Authors:** Mark B. Pinkham, Arjun Sahgal, Andrew P. Pullar, Matthew C. Foote

**Affiliations:** 1 Department of Radiation Oncology, Princess Alexandra Hospital, Brisbane, Queensland 4102 Australia; 2 University of Queensland, Brisbane, Queensland Australia; 3 Department of Radiation Oncology, Sunnybrook Health Sciences Centre, University of Toronto, Toronto, Ontario Canada; 4 Queensland University of Technology, Brisbane, Queensland Australia

**Keywords:** Brain metastases, Melanoma, Radiosurgery, Gamma knife, Whole brain radiotherapy

## Abstract

The routine use of adjuvant whole brain radiotherapy (AWBRT) after surgery or stereotactic radiosurgery is now discouraged by a number of international expert panels. Three decades of randomised studies have shown that, although AWBRT improves radiological measures of intracranial disease control, the clinical benefit is unclear and it is also associated with inferior quality of life and neurocognitive function. The number of patients with melanoma in these trials was low, but data suggesting that treatment-related side effects should vary according to histology of the primary malignancy are lacking. For metastatic melanoma, the role of AWBRT to control microscopic disease in the brain is also a less relevant concern because systemic therapies with intracranial activity are now available. Whether AWBRT is useful in select patients deemed at high risk of neurologic death remains undefined.

We read with interest the article by Fogarty et al. debating the role of adjuvant whole brain radiotherapy (AWBRT) after surgery or stereotactic radiosurgery (SRS) for brain metastases [1]. They propose that withholding AWBRT ‘may not be in the best interests of patients, nor of health systems’ and that the American Society of Radiation Oncology (ASTRO) should retract its recommendation to discourage the routine use of AWBRT in this setting [2].

The management of any patient with incurable disease should focus on maximising quality and quantity of survival. Local therapy without AWBRT is now a standard treatment for patients with limited number of brain metastases and a more favourable prognosis. This recommendation is endorsed by multiple expert panels including ASTRO, National Cancer Comprehensive Network (NCCN) and the European Association of Neuro-Oncology (EANO) [3–5]. Nearly three decades of randomised studies have consistently shown that the omission of AWBRT after surgery or SRS is not associated with inferior survival or functional independence [6–11]. Although AWBRT improves radiological measures of intracranial disease control, this is of uncertain clinical benefit because most patients die from extracranial disease and AWBRT adversely affects quality of life (QoL) and neurocognitive function (NCF) [8, 11, 12]. Despite an increased need for radiological surveillance and salvage therapy at recurrence, a number of North American studies have concluded that SRS alone provides cost-effective care compared to SRS with AWBRT [13–15].

Many patients will experience acute side effects during or shortly after AWBRT including dermatitis, alopecia and fatigue. Neurotoxicity is traditionally said to be a late effect only observed in a minority of long-term survivors. However this is inaccurate because deficits in NCF can occur within 3–6 months from treatment [8, 11, 16–18]. NCF is important because it can predict QoL [19], functional independence and survival [16, 20]. Declining NCF increases caregiver burden [21] and impairs financial, work and social activities [22, 23]. Although NCF was not assessed in the largest randomised study of AWBRT ever performed, patient-reported cognitive function was analysed [9, 12]. In 341 patients randomised to observation or AWBRT after SRS or surgery to 1–3 brain metastases, AWBRT was associated with significantly lower mean test scores at 8 weeks and 12 months. AWBRT also resulted in inferior health-related QoL relating to fatigue, physical functioning and role functioning at various time points. The Alliance randomised study presented at American Society of Clinical Oncology (ASCO) 2015 mentioned by Fogarty et al. has now been published [11]. This trial is noteworthy because the primary endpoint was NCF at 3 months. Out of 213 participants with 1–3 brain metastases, deterioration in at least one NCF test was noted in 64% of patients receiving SRS and 92% receiving SRS with AWBRT. Statistically significant deteriorations were seen in immediate memory (8% versus 30%), delayed memory (20% versus 51%) and verbal fluency (2% versus 19%). Overall QoL (mean change from baseline −0.1 versus −12 points) and functional well-being (2.5 versus −22.3 points) were also superior at 3 months for SRS alone. Such differences persisted in the subset of 34 evaluable patients (16%) surviving at least 12 months from randomisation and reached statistical significance at various time points. These findings are consistent with a similar but smaller study by Chang et al. [8], which was potentially confounded by an unexpected imbalance in median survival between the two arms. Therefore, although AWBRT reduces the rate of intracranial failure, overall its deleterious effects on NCF are greater in the majority of patients than the deleterious effects of recurrence itself. Data to suggest that this treatment-related effect should vary according to histology of the primary malignancy is lacking.

Fogarty et al. state that AWBRT avoids a neurologic death. This was shown in two randomised studies [6, 9] but not confirmed in the others [7, 8]. In the meta-analysis by Sahgal et al., the risk of neurologic death with AWBRT was reduced from 30% to 25% [24]. Given the potential toxicities described, whether AWBRT is justified in up to 20 patients to prevent one additional neurologic death is uncertain. For many clinicians, this uncertainty has grown with the emergence of increasingly effective systemic therapies exhibiting potential intracranial activity. Consistent with this change, a more contemporary multi-institutional prospective series of 1194 select patients with up to 10 brain metastases treated with Gamma Knife SRS without AWBRT reported a neurologic death rate of only 8% [25].

We applaud the investigators of the ANZMTG 01.07 WBRTMel trial for their dogged determination to complete accrual. The trial hypothesis is that the addition of AWBRT will improve intracranial control and survival without significant impairment of QoL or NCF. The primary endpoint is distant intracranial control at 12 months and the protocol has been amended to permit AWBRT with hippocampal avoidance. Although Fogarty et al. believe that the ASTRO recommendation is negatively impacting on accrual to this trial, it is possible other factors have also influenced the willingness of patients and physicians to recruit to this study. Whether clinical equipoise truly remains or not because melanoma-histology specific data is sparse, a major concern remains the clinical relevance of the trial primary endpoint which is a radiological measure of disease. Interpretability of the data may also be limited because stratification for systemic therapy utilisation is lacking. Since this trial was designed, a number of targeted and immunotherapy systemic agents have become widely available for metastatic melanoma. Many are known to have activity in the brain [26, 27] although potential effects on QoL, fatigue and NCF are not well described.

Various subgroup analyses and retrospective series might suggest subgroups of patients that could benefit from AWBRT, but reports are contradictory [28–30] and high quality data is lacking. The effects of AWBRT on intracranial disease control, QoL and NCF are now clearly defined in an unselected population. Whether AWBRT can be used to control microscopic or occult melanoma in the brain is a less relevant concern nowadays because systemic therapy can be used. The role of hippocampal sparing AWBRT is yet to be defined in any population and the ASTRO recommendation should not preclude the proper assessment of new technologies in appropriately selected patients. However, optimal sequencing of treatment modalities is now a more relevant area of investigation rather than the effect of SRS and/or AWBRT alone. In this context, mixed-histology trials without stratification are no longer appropriate and enrichment for certain molecular subtypes are required. Multicentre efforts will be needed to permit statistically sound results within a reasonable time frame, thus providing data that remains useful and relevant to patients and their doctors.

## Abbreviations

ASCO: American Society of Clinical Oncology
ASTRO: American Society of Radiation Oncology
AWBRT: Adjuvant whole brain radiotherapy
EANO: European Association of Neuro-Oncology
NCCN: National Cancer Comprehensive Network
NCF: Neurocognitive function
QoL: Quality of life
SRS: Stereotactic radiosurgery
