# Supplementary material for: In response to Fogarty et al. and why adjuvant whole brain radiotherapy is not recommended routinely
Source: BMC Cancer. 2017 Nov 15;17:768. doi: 10.1186/s12885-017-3672-z (PMC5688708; doi:10.1186/s12885-017-3672-z)
Supplement: Related texts

# In reply to Pinkham et al.

**Authors:** Gerald B. Fogarty, Angela Hong, Vinai Gondi, Bryan Burmeister, Kari Jacobsen, Serigne Lo, Elizabeth Paton, Brindha Shivalingam, John F. Thompson

**Affiliations:** 5 Melanoma Institute Australia, Poche Centre, North Sydney, Australia; 6 0000 0004 1936 834X grid.1013.3 Sydney Medical School, The University of Sydney, Sydney, Australia; 7 Australia and New Zealand Melanoma Trials Group (ANZMTG), North Sydney, Australia; 8 grid.430785.d Trans-Tasman Radiation Oncology Group (TROG), Newcastle, Australia; 9 0000 0004 0380 2017 grid.412744.0 Princess Alexandra Hospital, Brisbane, Australia; 10 0000 0004 0389 8485 grid.55325.34 Oslo University Hospital HF, The Norwegian Radium Hospital, Oslo, Norway; 11 0000 0000 9687 807X grid.413645.1 Central Dupage Hospital Cancer Center, Warrenville, Illinois USA; 12 0000 0000 9209 0955 grid.412647.2 University of Wisconsin Comprehensive Cancer Center, Madison, Wisconsin USA; 13 0000 0004 0385 0051 grid.413249.9 Royal Prince Alfred Hospital, Sydney, Australia; 14 grid.419545.8 St Vincent’s Hospital Radiation Oncology Dept, St Vincent’s Clinic, Suite 708, 438 Victoria St, Darlinghurst, NSW 2010 Australia

We thank Pinkham et al. for their reply to our article [1]. In many areas we are in agreement. For example, we agree that adjuvant whole brain radiotherapy (AWBRT) without hippocampal sparing administered to patients with brain metastases can cause side effects including adverse effects on quality of life (QoL) and neurocognitive function (NCF). We also accept the evidence that most patients die from extracranial disease so that the impact of any radiotherapy (RT) to the brain should be measured in terms of palliative endpoints and not overall survival.

The thrust of our article, however, has been missed. To repeat it - even though 170,000 patients are diagnosed with brain metastases (BMs) in the United States every year, and are therefore possible candidates for studies assessing the value of AWBRT, in three decades only 774 patients had been enrolled in randomised controlled trials (RCTs) before the publishing of the American Society for Radiation Oncology (ASTRO) Item 5 of its “Choosing Wisely 2014” list. This recommended: “Don't routinely add adjuvant whole brain radiation therapy to SRS for limited brain metastases” [2].

Our experience has been that the ASTRO guideline has had a significant negative impact on accrual to the internationally accruing ANZMTG 01.07 WBRTMel trial [31]. This is an RCT investigating AWBRT following local treatment (surgery or stereotactic radiosurgery) in patients with one to three BMs from melanoma - one of the histologies that has consistently shown high rates of distant brain failure [28, 32, 33], and thus most likely to benefit from WBRT. This trial is the only single histology AWBRT trial so far attempted. To date WBRTMel has accrued 214 of a required 220 patients but recently accrual has diminished dramatically. Several aspects of this trial had already been reported prior to the publication of ASTRO guideline [31, 32]. Dr. Pinkham’s institution, from which 29 patients had been randomised to the WBRTMel trial, has not accrued a single patient since the guideline was published. The guideline should at least have added a caveat “except in the case of a clinical trial”.

There is an urgent need for more data, especially when a phase two trial investigating hippocampal-avoiding AWBRT (HAWBRT) showed significantly less NCF decline compared to historical controls [34]. We now need more randomised data to confirm the benefit of HAWBRT. It is important to note that hippocampal avoidance is allowed on WBRTMel and over 22 cases have had this form of treatment.

If the WBRTMel trial shows AWBRT in melanoma to be useless, then we will be amongst the first to recommend that it be abandoned. However, we contend that the procedure should only be abandoned only on the basis of adequate level one clinical trial evidence. We contend that the accumulation of more data is a wiser choice than abandoning AWBRT before conclusive RCT evidence is available, especially in melanoma.
